# The Force of Character

**Published:** 1886-12-20

**Authors:** 


### THE FORCE OF CHARACTER.

As a man nears the boundaries of Time it seems that the years pass with accelerated swiftness, and, often, he finds himself looking sorrowfully back upon the Past, because he has not done more good, and his heart yearns to accomplish all he possibly can to benefit Humanity before he finally and forever puts off the corruptible for the incorruptible. So, as I go down the decline of years, I feel more and more constrained to lift my voice, however feebly, in advocacy of all that ennobles and truly greatens my fellow-creatures, especially the young men of the noble profession whose unworthy representative I have been from my early youth.

With this object in view, I hope my professional readers will permit me, from time to time, to briefly discuss, in the editorial pages of the RECORD, some thoughts upon subjects that my long experience has taught me will make my fellow-man better, happier, and more honored and beloved in whatever sphere of life he may move.

It is conceded by all thinking men that true greatness must be built upon Goodness, and without this solid basis the most dazzling structure of fame or reputation is insecure, and at any time may be overthrown by the breath of the world's condemnation. Goodness gives its tone, its unmistakable ring, to a man's principles; his principles form his character, mold his life, and fashion the weapons with which he will carve out his career in coming years.

Young men especially should never forget that it is of very great importance to them to enter upon the active duties of life with true exalted notions of Character, and that the living out these exalted ideas will bring happiness, honors, dignity, legitimate prosperity, and ultimate success. A man, whatever may be his profession, who is "great in goodness and good in greatness" is no less than a benediction to every soul with which he comes in contact, and by the immutable law of reactionary force, as he gives of himself to bless his fellow-man, he receives in return perhaps a hundred fold.

It is said that "there are two principles by which men act upon the minds of their fellow-men, to wit, power and influence. Power is mostly exerted in the shape of authority, and is limited in its sphere of action. Influence has its lever in human sympathy, and is as boundless in its operation. When rightly directed, it is, in its nature, its action and results as superior to the other as the spiritual to the animal—as mind to matter. If there were any doubt which of these principles most contributes in the formation of human character, we have only to look around us. We see that Power, while it regulates men's actions, cannot reach their opinions; it cannot modify disposition, nor implant sentiment, nor alter character. All these things are the work of Influence. Men frequently resist Power, while to Influence they yield an unconscious acquiescence."

It has been truly said that a man achieves his greatest triumphs, not by express commands but by a thousand indirect influences emanating from the spirit of those rather than from the letter. Truth, Consistency, Simplicity, and Benevolence or Love are qualities especially needed in the character which is to exert a wholesome and happy influence upon others. Truth engenders faith and trust; "Consistency of character is consistency of action with principle, of manner with thought, of self with self. It is moral repose; it invites confidence; it awakens veneration for the source of all virtue and a trust in the origin of all truth." Simplicity, or single-mindedness, is the opposite of Hypocracy. It never assumes to be what it is not; it never dissembles nor makes a man utter sentiments as his own, which his heart and conduct do not approve. Love or benevolence is the mainspring of all noble character. It is the fulfilling of the Divine law—that inspiration of every code, moral or civil, that has incorporated in its structure, the well-being of our fellow-men. In every condition, however humble in life, there are opportunities for its exercise, and it is this that gives to a man's life and character the delicate aroma of a fragrant flower.

Among the duties of a physician there is ample scope for the expression of this Divine feeling of the heart, and I am glad to know that in the medical fraternity there are recorded so many instances of benevolence, love, and self-devotion to the cause of Humanity. The profession of medicine itself is God-like in its character, and cannot we—old, middle-aged, and young—endeavor to make our own character beautifully harmonious with that of our profession, and have our hearts so full of good and great purposes, our lives so illumined with noble deeds, that when, at last, we are called up higher, our memory on earth will be like a "Glorious sunset that's golden to the last, and when 'tis over shines in the witnesses it leaves behind?" T. S. P.

